# A patient‐led, peer‐to‐peer qualitative study on the psychosocial relationship between young adults with inflammatory bowel disease and food

**DOI:** 10.1111/hex.13488

**Published:** 2022-04-05

**Authors:** Jenna Rines, Kim Daley, Sunny Loo, Kwestan Safari, Deirdre Walsh, Marlyn Gill, Paul Moayyedi, Aida Fernandes, Nancy Marlett, Deborah Marshall

**Affiliations:** ^1^ Patient and Community Engagement Research (PaCER) Calgary Alberta Canada; ^2^ Department of Community Health Sciences University of Calgary Calgary Alberta Canada; ^3^ BC Support Unit/AHSN Vancouver British Columbia Canada; ^4^ Vancouver Island Health Authority Victoria British Columbia Canada; ^5^ BC Children's Hospital Research Institute Vancouver British Columbia Canada; ^6^ Department of Pediatrics University of British Columbia Vancouver British Columbia Canada; ^7^ IMAGINE SPOR Chronic Disease Network Hamilton Ontario Canada; ^8^ Faculty of Health Sciences McMaster University Hamilton Ontario Canada

**Keywords:** Crohn's disease, diet, food, inflammatory bowel diseases, patient‐led research, psychosocial, ulcerative colitis

## Abstract

**Background:**

Inflammatory bowel diseases (IBDs) are chronic gastrointestinal diseases that negatively affect the enjoyment of food and engagement in social and cultural gatherings. Such experiences may promote psychosocial challenges, an aspect of IBD often overlooked and under‐supported in clinical settings and research.

**Objectives:**

This study explored the psychosocial experiences that young adults with IBD have with food via a qualitative patient‐led research process.

**Methods:**

Trained patient researchers conducted this study by engaging peers via semi‐structured interviews and focus groups in a three‐step co‐design process. Participants (*n* = 9) identified the research topic (SET), explored the topic and identified emerging themes (COLLECT), refined themes and made recommendations for healthcare system change (REFLECT).

**Results:**

Themes that emerged included: ‘Experimenting with Food’, ‘Evolution Over Time’, ‘Diet Changes are Emotional’ and ‘Role of Stigma’. Participants identified the significance and frustrations of repeated testing and experimenting with food compatibility, and noted nuances in food relationships as they gain knowledge and experience over time. They emphasized the importance of maintaining a sense of hope throughout and wished to impart this to newly diagnosed patients.

**Conclusion:**

Participants experience numerous psychosocial challenges as they strive to manage their diet, noting gaps in support available from IBD practitioners. Participants made practical recommendations for healthcare system change to improve patient outcomes, highlighting the importance of sharing stories and collaboratively including patients in the development of new services and protocols. Authors recommend further research in this area to build a body of knowledge and support that helps IBD patients maintain hope while navigating challenges with food.

**Patient or Public Contribution:**

The first four authors on this paper were the lead researchers in this study's design and analysis and identify as patients; they conducted the research with this identity at the forefront following a peer‐to‐peer research model. These authors were mentored by patient researchers who also contributed to the manuscript, and the research process itself was co‐lead and directed by other patient participants and consultants. Results and recommendations coming from this paper came directly from patient participants.

## 1 INTRODUCTION

Inflammatory bowel diseases (IBDs), which include ulcerative colitis (UC), indeterminate colitis, and Crohn's disease (CD), are chronic inflammatory diseases of the gastrointestinal tract.^1^ Patients suffering from IBD can experience a host of physical symptoms including, but not limited to, chronic diarrhoea, rectal bleeding, cramping, abdominal pain, fever, weight loss, nausea and vomiting.^2^, ^3^ Canada has one of the highest rates of IBD in the world, with over 270,000 people living with IBD; this number is projected to increase to over 400,000 people, or 1% of Canadians, by 2030.^4^, ^5^

Diagnosis of IBD typically occurs in adolescence and in adults 20–30 years of age.^4^ The cause of IBD is unknown but it is likely to be multifactorial.^6^ It is widely believed that the environment, which encompasses factors such as stress, plays an important role in disease aetiology and progression.^7^ Patients consider diet to be very important as an aetiological factor in IBD, although this relationship continues to be unclear and no single dietary intervention is effective in all patients. After hearing from patients via narrative interviews, Palant et al.^12^ note that diet is ‘often the first behavioural factor that is manipulated by individuals with IBD after the onset of symptoms’ and feelings of uncertainty and constraint impact their experience. As a chronic and invisible group of conditions, people with IBD are also burdened by extensive non‐medical problems, including those related to stigma as well as comorbid mental health and psychosocial issues.^13^

Although research continues to investigate the role of food in IBD symptomatology, treatment and recovery, it is crucial that work is also done to further understand the patient experience with dietary concerns.^14^, ^15^ Patient‐led research can help healthcare professionals understand the depth of patient experiences that are not currently addressed by the medical system, especially as it relates to psychosocial challenges.^16^, ^17^ There has been some qualitative exploration into the significance of food for people living with IBD, although it is noted that ‘the psychosocial impact of dietary beliefs and behaviours has been a neglected area of IBD research’.^18^ To our knowledge, there is also a lack of patient‐led research investigating the relationship between IBD and food.

Researchers' awareness of the typical age of IBD diagnosis and their own lived experience informed the age group selected for participation in this study. Young adults diagnosed with IBD recall early experiences with the diet and are old enough to make and reflect on their decisions and experiences about food. This is as opposed to paediatric patients for whom an adult is usually responsible for organizing and preparing meals on their behalf, or older adults whose recollection of earlier diet experiences may be less clear. Young adulthood is also a time when the psychosocial burden in patients with IBD tends to be high.^13^ In addition, to the researchers' knowledge, there is a lack of qualitative research focusing on the young adult IBD population as it pertains to experiences around food. For these reasons, this study seeks to better understand the complex relationship young adults with IBD have with food, as well as the psychosocial factors that impact this relationship, via a qualitative patient‐led research process.

## 2 MATERIALS AND METHODS

### 2.1 Approach

This study was carried out by patient peer researchers who were interns of the Patient and Community Engagement Research (PaCER) Programme, a partnership between the Cumming School of Medicine at the University of Calgary and the Strategic Clinical Networks of Alberta Health Services. It was conducted in compliance with the ethical standards of the University of Calgary Conjoint Health Research Ethics Board, CHREB Ethics ID: REB190989.

**Figure 1:**
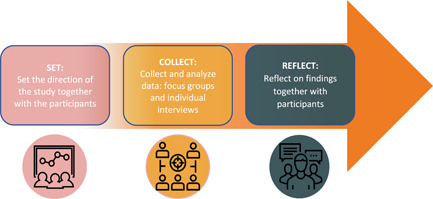
Outlines the engagement strategy used in the Patient and Community Engagement Research method. Research is conducted with patients in three distinct phases: SET, COLLECT and REFLECT

This qualitative study used the PaCER methodology (Figure 1) in which the researchers draw on their experience as patients to engage participants in a participatory peer research process. The engagement strategy, using SET, COLLECT, REFLECT, as demonstrated by Marlett et al.^19^, ^20^ and others,^21^, ^22^ ensures that participants are fully involved as consultants and co‐researchers throughout the research project, using the patient's voice to address their own needs and concerns. Participants involved in the research process are made aware of the PaCER engagement strategy, as well as the researcher's status as patient peers.

The SET stage is the initial exploratory phase intended for patient consultants to co‐design the study with researchers and identify the topics or concerns of most interest to patients. This information is used in the ethics proposal and guides the research into COLLECT. The COLLECT stage focuses on the topic most important to participants using iterative cycles of collecting and analysing data captured during semi‐structured interviews and focus groups. The REFLECT stage brings together participants from previous stages of the research, as well as others interested in consulting about the findings, focusing on important aspects of emerging themes and recommendations for healthcare system change.

### 2.2 Participants

Participants (*n* = 9) were individuals between the ages of 18 and 35 who self‐reported a diagnosis of IBD, including CD, UC, or indeterminate colitis. Participants were recruited via purposive and snowball sampling methods, such as through IBD social media accounts (Twitter, Facebook, Instagram), study website, word of mouth, personal contacts and posters in gastroenterology clinics. Participants contacted a member of the peer research team via email to express interest in the study, and researchers used predetermined inclusion and exclusion criteria to ensure participants were appropriate for the study. To participate, patients had to be between 18 and 35 years of age, self‐identify as having a diagnosis of IBD, have sufficient fluency in English to participate in interviews or focus groups and live in Canada. Patients could not participate if they had a close personal relationship with any member of the research team that may have influenced their ability to refuse participation.

### 2.3 Data collection and analysis

Patient participation across the three stages of the research engagement strategy can be seen in Table 1. In the pre‐study SET co‐design stage, researchers engaged six patients as consultants, representing the larger IBD patient community, in an online focus group (see Appendix SA for focus group guide). In the focus group, researchers brought potential topic areas based on their lived experience, as well as their knowledge of priority issues for the IBD community. Patients were asked to identify the most important topics of research relating to their experience with IBD, and they helped to set the direction of the research, as well as advised on data collection and recruitment methods. Information was captured as virtual flip chart notes, and patients had the opportunity to edit and prioritize ideas.

**Table 1:** Patient participation

Stage	Number of participants	Semi‐structured interviews/focus groups
SET co‐design—focus group	6	1 Focus group
COLLECT—focus group^a^	4	1 Focus group
COLLECT—semi‐structured interviews^a^	4	4 Semi‐structured interviews
REFLECT—focus group^a^, ^b^	5	1 Focus group

*Note*: Nine people participated in the study in total with three people participating in more than one stage of the study.

^a^ Only participants from COLLECT and REFLECT provided demographic information.

^b^ One participant from SET co‐design participated in the REFLECT focus group and provided demographic information.

It was at this stage where interest in the relationship between diet and IBD arose, and discussion identified the complex psychosocial issues related to food. This was the topic of most importance to patient consultants at this stage and researchers were urged to move forward with this topic. This information, along with a review of available literature on the patient experience with food and IBD, informed the ethics proposal required to formally initiate the research study, which was submitted to the University of Calgary Conjoint Health Research Ethics Board.

The COLLECT stage marked the beginning of data collection. Seven participants engaged in a 3‐hour online focus group and/or a 1‐hour online semi‐structured interview to explore their psychosocial experience with food while living with IBD (see Appendix SB for semi‐structured interview questions and story analysis guide). Following a similar format to the SET focus group, researchers opened the COLLECT focus group by asking participants to share their experience of the psychosocial relationship of food and IBD. From there the discussion was an open format with researchers asking clarification questions and facilitating to ensure all participants were able to share. Participants also took part in a basic sorting process of their data by identifying primary content areas to help generate initial codes via the electronic flip charting process capturing data as incidents. As in SET, participants were also invited to edit, add comments and expand upon flip chart notes. The semi‐structured interviews were captured using written notes and story templates.

Following an inductive thematic approach, analysis of focus group data began with all four researchers individually coding, organizing and tabulating the flip chart data into themes, and then comparing findings as a team.^23^ After the focus group was completed, semi‐structured interviews were then conducted by researcher pairs (see Appendix SB) who shared facilitation and notetaking responsibilities to promote thorough data collection. Both interviewers rotated asking questions and had equal opportunity to build rapport with the participant during the interview. Given that the interview was conducted virtually, notetaking did not appear to affect rapport‐building. Data were individually coded and analysed using a story analysis guide (see Appendix SC) before being reviewed and compared together by the interview pair.^19^ As with all data, it was then reviewed by the whole research team, which lent additional nuance to themes formed at this stage.

Researchers also reflected on their own lived experience as patients, contributing to the analysis. Memoing was an important part of the data collection and analysis process at all three stages, and researchers kept field notes on their observations as well as their own reflections as a patient going through this process. Once the final COLLECT interview was conducted, researchers' judgement confirmed that the interview did not produce any new themes or sub‐themes and confirmed data saturation had been reached at this point in the process.^24^, ^25^ Reaching saturation of themes and sub‐themes allowed researchers to move forward into REFLECT.

During the REFLECT stage, five participants reviewed, renamed, and redefined themes organized by researchers collectively in a focus group. Sub‐themes also emerged during this stage, and participants suggested ways to make use of the knowledge and stories obtained during the study. Recommendations for healthcare system change were collected as part of the data collection process, which is shared below. Researchers memoed, collated participant feedback and reviewed results during team discussions to ensure there were no new emerging themes.

Data collection occurred between October 2019 and January 2020. Each stage of the research was led by patient peer researchers and co‐designed with participants, and all four researchers were involved in conducting the focus groups and semi‐structured interviews. Data collection, analysis and memoing were performed in iterative cycles, and at least bi‐weekly discussions among team members informed planning and next steps. All stages of data collection were audio‐recorded, and process recordings were completed by a member of the research team. Process recordings were typed observations separate from flip chart notes and focused on the interactions, emotions and behaviours of participants during each data collection event. These products were timestamped and used by researchers to deepen their understanding of the data and research process.

### 2.4 Research credibility and trustworthiness

The success of a patient‐led qualitative research study rests on its implementation of effective strategies for iterative data collection and analysis. For this study, these strategies included consultations with patient participants and a research mentor who was in attendance during virtual focus groups as an observer and backup notetaker to ensure data accuracy in the potential event of technology failure. Given that the lead researchers were interns while conducting the study, this study mentor was assigned by the programme faculty to supervise the team's work. They also provided strategies for transparent and rigorous methodology during debrief meetings where they challenged researchers' modes of thinking to reveal any potential biases.^26^, ^27^, ^28^, ^29^ This supported reflexivity, credibility and trustworthiness of the research process.^26^, ^27^, ^28^, ^29^, ^30^ The research mentor also identified as a patient, was trained in the PaCER Programme and had previous experience conducting IBD peer‐to‐peer qualitative research. Iterative questioning was used in each data analysis cycle and included consultant and participant feedback via focus groups and individual interviews, which informed the next stage of the research. To promote transparency and reliability, researchers used strategies such as memoing, engaging in constant reflection and participating in regular team debriefs to challenge modes of thinking and reveal any potential biases.^26^, ^27^, ^28^, ^29^

The research team was composed of patients, reducing the power imbalance between researcher and participant. This enabled more in‐depth, productive and rich storytelling of patient experiences.^31^, ^32^ Three out of four of the research team members were young adults living with IBD, who provided helpful insight and potentially strengthened the research process through reflection on their lived experiences in the context of a ‘shared patient identity’ and participant findings.^20^, ^33^ At the same time, given this insider perspective, implementation of strategies that helped the team reflect and set personal boundaries such as memoing and team debrief meetings was important to minimize bias and avoid the influence of individual lived experiences.^20^, ^31^ Learning about researcher reflexivity via the PaCER Programme at the same time as conducting the study helped to keep these issues top of mind for the researchers.^20^ One member on the research team did not have IBD but lives with a chronic illness; this was beneficial to keep the team accountable and helped to challenge the team in considering multiple points of view. Before this study, the researchers had no experience conducting qualitative research, although one researcher had academic involvement with quantitative research and two others were involved as patient partners in their provincial patient‐oriented research units. Two team members were healthcare professionals, which was another level of identity that both deepened understanding of patient healthcare experiences and also required additional layers of reflection.

## 3 RESULTS

Participants were between 25 and 35 years old and ranged between three and 16 years of living with IBD (see Table 2 for additional detail). Most participants were female (*n* = 6), had not had surgery (*n* = 6) and lived with a diagnosis of CD (*n* = 6). At the time of the study, six out of nine total participants lived in Ontario, three participants lived in British Columbia, Alberta and Nova Scotia.

**Table 2:** Participant demographics

	Age^a^ (*n* = 9)		Diagnosis (*n* = 9)		No. of years since diagnosis (*n* = 9)				
	24–29	30–35	Crohn's disease	Ulcerative colitis (UC)	<5	5–10	11–20	>20	Unknown
M (*n* = 3)	2	1	2	1	0	0	1	1	1
F (*n* = 6)	2	4	4^b^	3^b^	1	1	3	0	1

*Note*: Demographic information available only for COLLECT and REFLECT participants.

^a^ No participants <24 years of age.

^b^ One patient self‐identified with both Crohn's disease and UC.

Iterative analysis of experiences shared by participants allowed for the co‐construction of four emerging themes: ‘Experimenting with Food’, ‘Evolution Over Time’, ‘Diet Changes Are Emotional’ and ‘The Role of Stigma’ (Table 3). Participants identified ‘Experimenting with Food’ and ‘Evolution Over Time’ as the two most important themes. Researchers also identified several sub‐themes. The last sub‐theme of each theme offers reflections on ways participants were able to reclaim or move forward in their relationship with food despite challenges. This will be explored further below.

**Table 3:** Themes and sub‐themes Identified in COLLECT and REFLECT stages

Themes	Sub‐themes
1. Experimenting with food	a. Trial and error
	b. Not black and white
	c. Weighing risks and benefits
	d. Developing knowledge and experience
2. Evolution over time	a. Starts with a lack of professional guidance
	b. Experimentation and independent research
	c. Seeking second opinions
	d. Acceptance and maintaining hope
3. Diet changes are emotional	a. Flares before and after
	b. Losing comfort foods
	c. Navigating social/cultural gatherings
	d. Guilt/burden of diet changes on others
	e. Reclaiming joy
4. The role of stigma	a. Support system (positive or negative)
	b. Judgement from others
	c. Justifying your diet
	d. Unsolicited ‘cures’
	e. Self‐advocacy

### 3.1 Experimenting with food

This theme describes the process of introducing and/or eliminating different foods to find a diet that brought participants closer to feeling well or more in control of an unpredictable disease, as illustrated below:

*Over time, you learn what food you can trust. Sometimes food will sort of break your trust over time as it is not black and white*. (P6 [Male, 28 years]‐RFG2)

*I think I had to find a way to feel more in control of it, and so that's really dictated how I eat, when I eat, who I eat with. It's all based on trying to feel like I can control the situation in a very uncontrollable situation*. (P8 [Female, 31 years]‐NI)

Four sub‐themes were identified pertaining to experimenting with food and are provided in Table 4 along with participant experiences.

**Table 4:** Experimenting with food sub‐themes

1a.	Trial and error	Experimenting with the quantities of foods consumed, the time of day to eat, where to eat and who to eat with to lessen the psychosocial stressors.
		‘Experience, that whole idea of failing forward ‐ trial and error, is very personalized, would give me hope, because the message that I got from professionals eventually was "we don't know, there's no one way to eat so figure it out"’ (P2 [Female, 29 years]‐RFG).
1b.	Not black and white	Experimenting with food is not a straightforward process. There are no hard or fast rules that would make it easy to know what foods to eat and what to avoid. ‘I weigh things, I read the ingredients, I know what's worked before… I don't think there's a hard black or white at all, because one (food) will work one day, and the next it's like “that's not good anymore”’ (P1 [Female, 33 years]‐RFG).
1c.	Weighing risks and benefits	Weighing risks and balancing those with the benefits of eating or not eating certain foods. Taking more risks in a familiar or comfortable setting in comparison to a public or unfamiliar setting. ‘A couple of times I have gone to family functions and I have said oh I will just eat it this once. Then a couple hours later, regretting it. I've learned that it's just not worth the sacrifice’ (P7 [Female, 35 years]‐NI).
1d.	Developing knowledge and experience	The role of independently experimenting with different foods in a trial‐and‐error method and conducting independent research is what increases this combination of knowledge and experience gained over time. ‘What did not work for me early on that works for me now, it came from time and experience. I can collect as much knowledge as I can, but until I have an experience, I don't know how well it will work for me’ (P1 [Female, 33 years]‐RFG).

### 3.2 Evolution over time

At the early stages of diagnosis many participants felt alone, lost, and hopeless due to a lack of professional guidance in relation to food and IBD:

*The message that I got from professionals eventually was ‘we don't know, there's no one way to eat, so figure it out’*. (P2 [Female, 29 years]‐RFG)

*I remember being very frustrated with food in general*. (P1 [Female, 33 years]‐NI)

Over time this set most participants in the direction of experimentation and individual research, and many sought a second opinion for advice from a variety of different professionals. Table 5 provides sub‐themes illustrating participant experiences related to the evolution over time concept.

**Table 5:** Evolution over time sub‐themes

2a.	Starts with a lack of professional guidance	The initial lack of support from professionals, especially when in the initial diagnosis phase of their IBD. Many participants noted being told that food did not play an important role in the progression of the disease, and to eat whatever they want. ‘GIs are generally like “Food doesn't matter, okay move on, let's find you a medication”. There's not a lot of time spent with patients talking about the role food plays in their life’ (P1 [Female, 33 years]‐NI).
		‘I felt like I was sort of alone in this journey of food because I find in terms of the healthcare system it's so not designed to deal with these types of things. Like from the GI point of view they're very like “What's going on in your gut?” and they usually tell you “No, food doesn't play a role”, but obviously it does’ (P8 [Female, 31 years]‐NI).
2b.	Experimentation and independent research	Independently experimenting with different foods and strategies to manage diet changes. Participants learned how to do this experimentation by independently researching through books, internet, blogs and other people with IBD. ‘I got zero support from my gastroenterologist and that's when I knew I had to do my own research’ (P2 [Female, 29 years]‐CFG).
2c.	Seeking second opinions	Seeking additional advice from dieticians, naturopaths, mental health professionals or with other gastroenterologists to improve their relationship with food and IBD. ‘I saw a naturopath and she helped me realize that a lot of the foods I was eating were disturbing my gut, more so than it needed to be. So, it changed my relationship with food because I felt like I needed to re‐learn my new version of healthy’ (P5 [Female, 26 years]‐NI).
2d.	Acceptance and maintaining hope	Participants noted the tools, information and experience they gathered seemed to enhance and deepen their relationship with food. Arriving at this state of acceptance was a lengthy process, but maintaining hope throughout, especially during the most difficult moments, helped them keep going. ‘Initially though, you're like “oh this sucks, I miss these foods”, but then you go through that whole phase of just acceptance and kind of troubleshoot your way… For me, yeah I think about food all the time, but it's just part of my life, and I've chosen not to be a victim anymore, and I just do what I know is going to feel best’ (P2 [Female, 29 years]‐RFG).

### 3.3 Diet changes are emotional

This theme described the emotional impact of food restrictions, diet changes, inability to eat, loss of comfort foods and the overall lack of guidance from professionals:

*I loved pasta, like that was one of my go‐to meals. So losing it was pretty rough. I was excited to have it back but pretty nervous and anxious about trying it again, especially if I did find out that it was something that made me feel horrible. That would have been very devastating*. (P1 [Female, 33 years]‐NI)

Participants described feeling not in control of their experience, exhausted from meal planning, isolated from some social gatherings and feeling like a burden to others. Some participants noted that they developed unhealthy eating habits, such as binge eating, to cope with the emotional impact of diet changes. These experiences are captured in the sub‐themes explored in Table 6.

**Table 6:** Diet changes are emotional sub‐themes

3a.	Flares before and after	How their relationship with food changes during times of flares in comparison to times of feeling well. Foods that were once ‘safe foods’ became ‘trigger foods’ and were no longer trusted. ‘I feel like every time after a flare there's a new food that I can't tolerate’ (P3 [Female, 32 years]‐CFG).
3b.	Losing comfort foods	Many foods that a person would turn to during emotionally stressful times were often ‘trigger foods’ for our participants. ‘I mean, for me, all of a sudden not being able to eat something that I've liked for so long, it's really depressing, really – something that was comfort food all of a sudden becomes the “enemy” ‐ “what am I going to eat?”, it becomes a source of frustration’. (P1 [Female, 33 years]‐CFG). One participant described feeling a ‘change of identity or loss of identity’ and the sadness surrounding that.
3c.	Navigating social/cultural gatherings	The emotional and psychosocial challenges experienced when attending social or cultural gatherings where food is a highlight. ‘I don't know what the cake tastes like that we are serving to our guests. You're very invested into your own wedding. You want to know what experience the guests are going to have. So, in a moment like that, I felt a little left out. I would say to my mom and dad “describe everything, what does the cake taste like?”’ (P5 [Female, 26 years]‐NI).
3d.	Guilt and burden of diet on others	Feelings of guilt or being a burden when asking family or friends to make accommodations for food restrictions. ‘I do still have a little bit of guilt associated with knowing that both of us… you know he has IBD by association, so when it comes to us making plans, we do have to consider what I can eat and when I can eat it’. (P3 [Female, 32 years]‐CFG). Participants described feeling like a burden when asking others to make different meals for them at social and/or cultural gatherings or asking to eat at specific restaurants that offer food they could eat.
3e.	Reclaiming joy	Diet changes caused many emotions, such as sadness, frustration and fear; some participants described the joy of food being taken away entirely. Participants discussed their process of finding ways to reintroduce pleasure and enjoyment into their relationship with food. This reclamation process often seemed to involve redefining how they viewed or interacted with food. ‘Before I saw the dietician, I was eating out of necessity but not really enjoying the food, because it was always stressful because there was always discomfort’ (P7 [Female, 35 years]‐NI).

### 3.4 The role of stigma

Primarily, stigma was defined as the judgement participants received on what they were or were not eating because of their IBD. Experiencing judgement and the offer of unsolicited advice about ‘cures’ left participants sometimes feeling as though their IBD was something they caused or could have prevented:

*Everybody's mindset is ‘well stop eating this’, or ‘if you have that then you're cured’*. (P4 [Male, 33 years]‐CFG).

Five sub‐themes were identified and matched with participant experiences in Table 7 below.

**Table 7:** The role of stigma sub‐themes

4a.	Support system—positive or negative	Support from medical professionals, family, friends, co‐workers, blogs, support groups or others with IBD. ‘Going to the local support group for people living with IBD has been really helpful in terms of coping… there's a lot of good experience around that, and a hopefulness’ (P1 [Female, 33 years]‐NI). Unfortunately, some participants described scenarios where friends, family members, and even spouses were unsupportive, thereby intensifying their experience of food‐related stigma.
4b.	Judgement by others	Judgement by others for what foods they were or were not eating. ‘There's always a friend that makes comments about food or something like that. Now that I am taking food very seriously as an intuitive approach, if anyone says any comments about “you should eat this” or “you shouldn't eat that”, it kind of makes me back off because it makes me feel like “well I am doing what my body is asking and it's none of your business”’ (P5 [Female, 26 years]‐NI).
4c.	Justifying your diet	Feeling the need to justify their diet to mitigate judgement from others. ‘I'm pretty open. It took me a few years. I was always very secretive about everything and now it's just, I would tell them exactly. I would say, I have these restrictions because I have colitis’ (P7 [Female, 35 years]‐NI).
4d.	Unsolicited ‘cures’	Advice from others on how to cure their IBD by eating certain foods or following diet fads. 'A lot of people were like “well maybe you should change the way you eat entirely, you should go back to eating a more Western diet because you will probably get better” and I was like, “I don't think that's going to” be the way that works’ (P1 [Female, 33 years]‐CFG).
4e.	Self‐advocacy	The importance of feeling empowered to prioritize their well‐being above all else, citing that they know their bodies and food needs intimately so that they can return to daily activities important to them. They become more comfortable with being upfront and open about their disease and diet restrictions to self‐advocate in the face of judgement and stigma. ‘I think it does have a lot to do with a sense of empowerment over living with the disease. I choose not to eat that food because I know I feel awful, or I know it's something that makes me feel like I have to go home after I go out for dinner instead of go to a show’ (P1 [Female, 33 years]‐NI).

### 3.5 Moving forward

*It's much better now. Now I've adjusted to that way of being, and so I don't think too much about it, when it comes to eating differently*. (P1 [Female, 33 years]‐NI)

**Figure 2:**
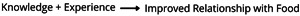
Improved relationship with food. The figure depicts a formula demonstrating the process of participants' improved relationships with food, which was the sum of their knowledge and experience

As mentioned above, participants discussed how knowledge and experience help them to continue to move forward with their relationship with food. This progress helps patients reach a state of increased acceptance and hope for future experimentation. Based on these ideas, it was apparent that participants were attributing their improved relationships with food to the sum of their knowledge and experience, as illustrated in the formula below in Figure 2.

Further analysis of themes and sub‐themes revealed that participants focused very strongly on the role of knowledge and experience gained over time as being a protective factor in navigating many of the psychosocial challenges of experimenting with food. This was especially noticeable in the sub‐themes ‘Developing Knowledge and Experience’, ‘Acceptance and Maintaining Hope’, ‘Reclaiming Joy’ and ‘Self‐Advocacy’.

## 4 DISCUSSION

Although there was significant participant discussion of their distress when things did not go well, it is noteworthy that participants shared, without prompting, how they are also thriving despite their challenges with food. Many participants had arrived at a place of acceptance of the experimentation process and trusted in their ability to do their own research and to know what is best for them. This point of acceptance and maintaining hope was a product of their knowledge and experience gained through the experimentation process. Arriving at this state of acceptance was a lengthy process (made longer by lack of professional guidance), and participants noted that maintaining hope throughout, especially during the most difficult moments, helped them keep going:

*There are a lot of negatives, and I think that needs to be conveyed to doctors, especially those who are newly diagnosed, but I think it's equally important to put out there that there is a process. Everyone will fall on a spectrum… How to navigate and learn and grow so you're not a victim to this*. (P2 [Female, 29 years]‐RFG)

Participants wished for this information to be shared broadly, especially with newly diagnosed patients. For the researchers, these stories reinforce the remarkable resilience of patients as they navigate their perceived gaps in care of the healthcare system. Researchers of this study acknowledge resource limitations within healthcare systems and empathize with the serious pressures healthcare providers experience. It is unrealistic to expect healthcare professionals to have all the answers or develop comprehensive tailored interventions for each patient. However, since many patients are going to be experimenting with their diet and limiting the intake of certain foods, an important first step will be facilitating a supportive space where patients are heard, and difficulties are normalized in the context of living with a challenging chronic illness.

Recent literature maintains that diet ‘plays a key role in the complex aetiology and treatment of IBD’, yet current Western medicine lacks emphasis on dietary counselling and resources.^34^, ^35^, ^36^ Our study substantiates these findings, as well as that the psychosocial challenges of food heavily influence the disease experience of patients with IBD.^12^, ^14^ The fact that almost all social events centre around food was an important factor discussed by participants. Food is more than just physical sustenance, having cultural, social and psychological significance.^37^

Peters et al.^34^ created a food frequency questionnaire specifically used to assess dietary intake for patients with IBD, which when included as part of gastroenterologists' clinical assessment would likely ‘avoid unnecessary self‐prescribed dietary restrictions’ and unsafe experimentation.^35^ Palant et al.^12^ highlight that advising about food and eating also includes supporting strategies to prevent social isolation, a key finding of this study. This would especially support patients early on in their IBD journey to understand how the role of stigma, stress and other psychosocial challenges may impact their navigation of relationships and social events.

This patient‐led study provides a much‐needed perspective of the psychosocial challenges of food and IBD in healthcare research. It also identifies clear perspectives that patients have while moving through diet‐related challenges, ranging from despair, to resourcefulness, to hope. Participants in this study described having a complex psychosocial relationship with food as they managed constantly changing food tolerances and setbacks, emotional distress, social isolation and stigma. Although participants found their way to knowledge and experience, for the most part on their own, they made it clear that they wished they had support from professionals to make this journey smoother. Without guidance from healthcare professionals to navigate issues around food, patients are left with the accompanying psychosocial challenges, confusing information and finding ways to manage on their own.

**Figure 3:**
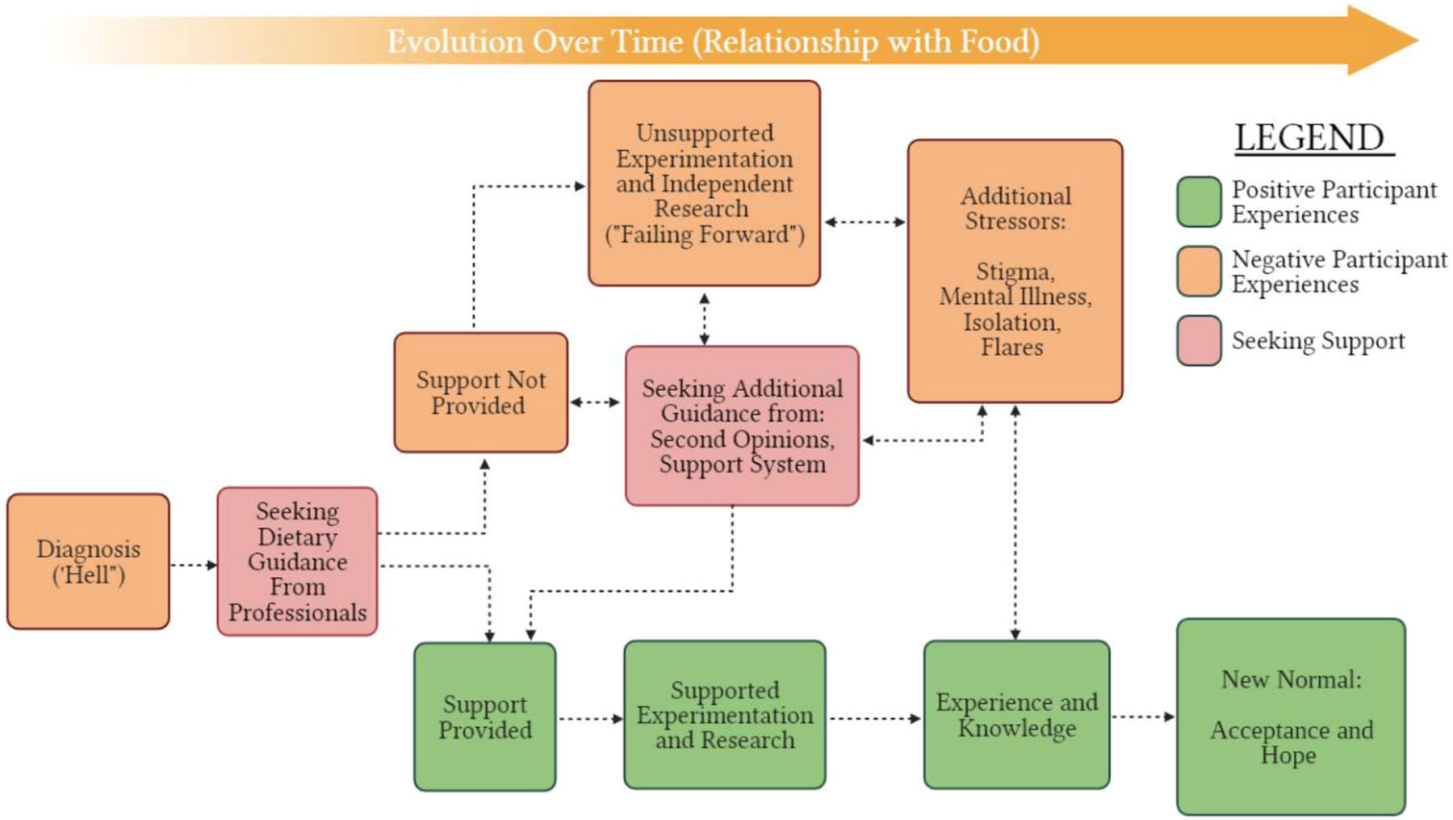
The evolution of participant experience with food over time. This figure is a flow chart that researchers used to map out participant accounts of their journey with food. The flow chart begins on the left with ‘Diagnosis’ and follows the arrows moving right based on their positive or negative experiences within the healthcare system. Even if participants' experiences started out as negative, all participants described arriving at a ‘New Normal’ at the bottom right of the flowchart

Researchers mapped out participant accounts of their journey with food in the form of a flow chart (Figure 3). Although participants emphasized that this is not a ‘one size fits all’ process, the flow chart illustrates the pathways that patients may experience as they seek support with diet challenges. Participants discussed negative experiences starting right at diagnosis and suggested that some of the stigma, mental illness, isolation, flares or stress could have been avoided if the health care system supported their experimentation with food earlier on. To participants, the evolution of their relationship with food over time was important to highlight their progress and demonstrate to newly diagnosed patients that there is hope for change in the future.

## 5 STRENGTHS AND LIMITATIONS

A unique strength of this study is that all stages and aspects of this study were designed, performed and analysed by patients.^19^, ^20^, ^36^ As discussed, possible power imbalances were likely reduced given the researchers' explicit role as patient peers, providing opportunity for rich stories to be shared by participants. Researchers' own nuanced lived experience provoked a greater depth of understanding of the participants' experiences, as well as deeper reflection and analysis given their understanding of the issues discussed.^19^ Conducting the research remotely provided a greater geographical reach for patients across Canada to take part, as well as increasing accessibility for patients who may not have been well enough to participate in person. Researchers also conducted semi‐structured interviews in pairs and used a team approach to data collection, which may have improved the findings' quality, credibility and trustworthiness.^30^, ^33^, ^38^ At the same time, sharing responsibilities in the research process created opportunity for different interpretations of the data to arise due to researchers' personal lived experience with IBD. To mitigate these different interpretations, recordings and analysis of each interview were shared amongst all researchers to review. Awareness of potential bias supported more objectivity in analysis, and researchers minimized the influence of individual lived experience with regular meetings to discuss findings and engaged in individual reflection through memoing.

Being a qualitative study with a small sample size, participant perspectives discussed here will certainly not be reflective of all of the diverse experiences of IBD patients. It should be noted that there were disproportionately more participants identifying as female and living in Ontario, and all participants identified as ‘white’. All of these factors affect how participants navigate their regional healthcare system and are noted as limitations of the study. Researchers also faced technical difficulties and relied on participants' ability to manage the videoconferencing and virtual flip chart platform and their access to sufficient internet bandwidth. We offset some of these issues by (a) sending several communiques with instructions and offering support with technical issues, and (b) selecting user‐friendly technologies for access on computers and smartphones.

## 6 RECOMMENDATIONS

In alignment with our goal to give voice to patients living with IBD, recommendations listed below in Table 8 are those expressed directly by the study participants. The aim of sharing these is to promote a deeper understanding in healthcare providers and policymakers of the challenges patients with IBD face when it comes to food, as well as finding actionable ways to increase support for and with patients. Participants' recommendations cluster around addressing what the researchers have linked to three main sub‐themes: addressing the ‘Lack of Professional Guidance’, supporting ‘Experimentation and Independent Research’ and building a more reliable ‘Support System’.

**Table 8:** Patient participant recommendations

Corresponding sub‐theme	Recommendation from participants
2a. Lack of professional guidance	Create a centralized resource and referral website with facts and contact information of specialized IBD healthcare professionals to support diet and nutrition.
	Provide additional training to healthcare professionals regarding the psychosocial impact of food, its significance for symptoms and the emotional impact of this to help facilitate referrals to appropriate services and other professionals.
	Provide access to IBD case managers that help patients navigate the healthcare system, coordinate tailored dietary recommendations and referrals and to listen to their psychosocial experiences with food.
2b. Experimentation and independent research	Create a working group of healthcare professionals and patients to collaborate and determine clinical guidelines that help patients experiment with diet.
4a. Support system	Record and share patient stories about their experiences with food more widely to encourage increased awareness of issues and messages of hope amongst patients and IBD healthcare professionals (via social media, podcasts, websites, videos, webinars).
	Create more supports, like support groups and non‐judgmental spaces for patients to talk, feel less alone and isolated.

Abbreviation: IBD, inflammatory bowel disease.

To a reader unfamiliar with diet‐specific resources available to patients with IBD, these recommendations seem that they either should or already do exist. This is not surprising to the researchers of this paper. The takeaway message here is that regardless of whether these resources exist or not, to experienced patients, some with over 20 years of experience living with IBD, their experience of the service gaps are real. Therefore, continuing to increase access, awareness and support for patients as they experiment with diet is a must.

It is also noteworthy that the recommendations provided were focused on looking at ways to have a productive conversation between healthcare professionals and patients to promote system and practice change. Participants encouraged the implementation of dietary clinical guidelines as well as ways to share stories that seek to educate and advocate to prevent many of the negative experiences our participants faced in other patients. The research team recommends that further peer research in this area is conducted to build a body of practical knowledge that helps patients maintain hope while managing dietary concerns related to their illness.

## 7 CONCLUSION

This study shares perspectives of young adults with IBD about their psychosocial relationships with food, providing practical strategies and recommendations for healthcare professionals and healthcare systems more broadly to implement change. In the absence of change, patients will continue to experience avoidable barriers to their recovery and risk further physical, social and psychological harm.

As discussed, more patient‐driven and interdisciplinary research is needed to encourage meaningful change in healthcare systems and practice to better support patients' dietary experiences. We also suggest examining in greater detail the unique psychosocial impacts of food that might be present for specific IBD patient groups, such as those who live with an ostomy or pelvic pouch.

Patient‐led research is shedding light on areas for healthcare practice improvement, and it is important to recognize patients as partners in healthcare system transformation. Researchers of this study are engaging with academic, healthcare and patient peers about the results and recommendations, and it is hoped that through connection and collaboration, more accessible and effective healthcare can be possible for people with IBD.

## AUTHOR CONTRIBUTIONS

All authors have contributed to the conception and design of the protocol. Kim Daley, Sunny Loo, Kwestan Safari and Jenna Rines constructed the first draft of the article, which was significantly revised by all other authors. All authors have given final approval of the version submitted for publication.

## CONFLICTS OF INTEREST

Dr. Moayyedi holds the Audrey Campbell Chair in Ulcerative Colitis Research. Dr. Marshall holds a Canada Research Chair (2008–2018) and the Arthur J.E. Child Chair and receives travel reimbursement through Illumina for meetings of the Global Economics and Evaluation of Clinical Genomics Sequencing Working Group. Dr. Marlett, Dr. Marshall, Dr. Moayyedi, Aida Fernandes, Deirdre Walsh, Marlyn Gill, Kim Daley, Sunny Loo, Kwestan Safari and Jenna Rines have no conflicts of interest.

## ETHICS STATEMENT

This study was conducted in compliance with the ethical standards of the University of Calgary Conjoint Health Research Ethics Board. Ethics ID: REB19‐0989. Freely given informed consent to participate in the study was obtained from all individual participants included in the study.

## Supporting information

Supplementary Information

## Data Availability

The datasets generated during and/or analysed during the current study are available from the corresponding author on reasonable request. All materials have been provided to the University of Calgary for appropriate storage.
