# Supplementary material for: A patient‐led, peer‐to‐peer qualitative study on the psychosocial relationship between young adults with inflammatory bowel disease and food
Source: Health Expect. 2022 Apr 5;25(4):1486–97. doi: 10.1111/hex.13488 (PMC9327832; doi:10.1111/hex.13488)
Supplement: Supplementary file 1 — Supplementary Information [file HEX-25--s001.docx]

**APPENDICES**

**Appendix A: IBD National Co-Design Focus Group Guide**

**Videoconference via Jitsi Meet**

**April 28, 2019 - 2 PM to 5 PM EST**

**2:00 – 2:05 Greetings:**

Allow time for all participants to join

Deal with any technical issues participants may have (audio, video, etc.)

**2:05 – 2:15 Introductions:**

Notice of recording and option to withdraw

Introduction to the team

Introduction to PaCER methodology

Purpose of focus group

Confidentiality

Schedule and housekeeping

How to use Jitsi Meet

Participant introductions

**2:15 – 3:00 Open discussion:**

Starting question: Thinking about your own experience with IBD, and that of other people you might know or worked with or have experience with, what are some topics that you think are important to individuals with IBD?

Team facilitates discussion and takes notes via flip chart and process recordings

**3:00 – 3:25 Team presents additional topics from lived experience, consultation, and research:**

Relationships

Diet

Intimacy

Mental health

**3:25 – 3:30 Participant reactions to what has been shared so far:**

Anything missing?

Any questions?

**3:30 – 4:00 pm Break**:

During this break participants prioritize ideas using the electronic flip chart notes

Participants are encouraged to add comments and anything missing

Team provides instructions for the second half of session (ranking ideas and discussion)

**4:00 – 4:45   Open discussion on flip chart prioritization:**

Participants discuss what was most important to them, team facilitates discussion

Who do you think would be most interested in talking about this topic?

How should we go about finding participants interested in this topic?

What is the best medium to connect to this group?

Based on the discussion, what kind of research could come from this session?

**4:45 – 5:00 Wrap up:**

Thank you

Summary of discussion

Next steps in research process

## **Appendix B: Semi-Structured Interview Questions**

Based on results from the COLLECT focus group we wanted to explore in more depth several emerging categories. As a result, the interview included as a starting point the following guiding questions:

Tell us about the psychosocial impact that food has had on your journey with IBD (if any).

Tell us about your emotional experience with food in the beginning with IBD.

Tell us about the process of making diet changes or making adjustments – both recently and at the beginning of your diagnosis.

Describe how your mental health/emotional health was affected by these diet changes.

How did stigma impact your relationship/journey with food?

How has your relationship with food evolved over time? Describe this relationship.

How would you describe your process of “negotiating” with food?

## **Appendix C: Story Analysis Guide**

*Interviewers:*

*Interviewee:*
*Interview Date:*
*Topic: Psychosocial relationship with food*

| Title: A Peer-to-Peer Study on the Psychosocial Relationship Between Individuals with IBD and Food |
| --- |
| Context: |
| *Plot (triggers, events):* |
| *Outcomes/Consequences/Lessons (Researcher’s summary):* |
| *Storyteller's (participant’s) reaction to telling the story/what they learned:* |
| *Researcher’s reaction to the story, what researcher has learned:* |
